# Therapeutic Effects of Ethanolic Extract of *Polygonum limbatum meism* Against Reproductive Toxicity Induced by Cadmium in Male Guinea Pigs (*Cavia porcellus*)

**DOI:** 10.3389/fvets.2021.736836

**Published:** 2021-10-12

**Authors:** Brice Menkem, Bertin Narcisse Vemo, Megnimeza Martine Astride Tsambou, Tadiesse Lavoisier Fonou, Nguedia Arius Baulland Dongmo, Judith Laure Boufack, Margaret Mary Momo Chongsi, Augustave Kenfack

**Affiliations:** ^1^Department of Animal Science, Faculty of Agronomy and Agricultural Sciences, University of Dschang, Dschang, Cameroon; ^2^Department of Animal Science, Faculty of Agriculture and Veterinary Medicine, University of Buea, Buea, Cameroon

**Keywords:** cadmium chloride, male guinea pigs, oxidative stress, reproduction, *Polygonum limbatum meism*

## Abstract

This study aimed at evaluating the therapeutic effects of ethanolic extract of *Polygonum limbatum meism* (EEPLM) on the reproductive parameters of male Guinea pigs exposed to cadmium chloride. Thirty-six male guinea pigs were randomly assigned to six treatment groups (with six animals per group). Group 1 (DW) received distilled water orally; group 2 (Cd), negative control, was treated with cadmium chloride at a dose of 26.25 mg/kg body weight (bw); while group 3 (VitC), positive control, was given 26.25 mg of cadmium chloride/kg bw and 100 mg of vitamin C, and groups 4, 5, and 6 were treated, respectively, with EEPLM at doses of 50, 100, and 200 mg/kg bw in addition to cadmium chloride (26.25 mg/kg bw). After 90 days, all animals were sacrificed, and data related to reproduction, toxicity, and oxidative stress were collected. Results revealed a significant decrease (*p* < 0.05) of serum levels of creatinine, urea, alanine, and aspartate amino transferases in guinea pigs treated with cadmium chloride and EEPLM compared to the negative control group (Cd). The weight of the bulbo-urethral gland was significantly (*p* < 0.05) decreased in animals exposed to cadmium chloride and treated with vitamin C or EEPLM compared to the negative control (Cd). Guinea pigs orally receiving cadmium chloride and EEPLM showed significantly (*p* < 0.05) increased motility, sperm count, spermatozoa with entire plasma membrane, and percentage of normal spermatozoa with reference to the negative control (Cd). The serum level of testosterone increased insignificantly (*p* > 0.05) in animals given cadmium and EEPLM compared to the negative control (Cd). Animals co-administered cadmium chloride and EEPLM recorded a significantly (*p* < 0.05) reduced level of MDA, activities of SOD, and total peroxidases compared to the group that received cadmium chloride (Cd) only. In conclusion, cadmium chloride induced reproductive impairments by generating oxidative stress. However, the administration of EEPLM can mitigate these adverse effects due to its antioxidant properties.

## Introduction

Cadmium (Cd) is an environmental pollutant commonly considered as the most important toxicant for humans and animals, due to increasing levels in the environment because of industrial and agricultural practices (1). It is extremely hazardous to life and has been involved in historic poisoning episodes of human and animal populations. It is a serious lethal occupational and environmental toxicant, known for its high toxicity, which may affect living systems in various ways (2). Heavy metals such as cadmium generally show their harmful effects through reactive oxygen species (ROS) production or inhibition of antioxidant enzyme activities (3). Cadmium when absorbed after oral exposure accumulates in the liver and kidneys, where it induces injury including protein denaturation and formation of ROS, lipid peroxidation, tubular degeneration, tubular cell apoptosis, interstitial inflammation, and glomerular swelling (4). Cadmium exposure generates reactive oxygen species that affects the male reproductive system and deteriorates spermatogenesis, semen quality, sperm motility, and hormonal synthesis especially (5).

This is because cadmium, once in the body, enters cells through calcium channels inside which it is bound to metallothionein. When the intracellular synthesis of these metallothioneins is no longer proportional to the quantity of accumulated cadmium, the cadmium is found in the free state (cd^2+^) and thus generates disturbances leading to significant harmful consequences for the cell, in particular apoptosis and a significant production of free radicals (6).

However, several measures to neutralize the oxidative damage in animal organisms include the use of antioxidants. Antioxidant molecules, such as butylhydroxytoluene, salicylic acid, selenium, and vitamins E and C, are usually used (7, 8), but their high costs and their possibility to cause diseases like cancers constitute limits for their use nowadays. However, the use of medicinal plants to reduce oxidative damage has increased worldwide. This is because they are rich in many natural antioxidants like phenols, flavonoids, terpenoids, and xanthons, and especially due to their low toxicity (9). *Polygonum limbatum meism* is one of these medicinal plants with compounds that are possibly effective against heavy metals toxicity (10). The *Polygonum* genus contains 300 species found all over the world. They contain diverse pharmacologically active constituents with various properties (11). These species are predominantly herbs, found in tropical and temperate regions (12). This species is well-known for producing a wide variety of secondary metabolites including flavonoids, triterpenoid, anthraquinones, coumarins, phenylpropanoids, lignans, sesquiterpenoids, stilbenoids, tannins, proteins, amino acids, and carbohydrates (10). Many of these *Polygonum* species and their active principles have been studied for pharmacological purposes. They have shown efficacy in the prevention and treatment of diseases such as cancer, malaria, gastric ulcers, and bacterial and fungal infections (10, 13). Therefore, from these antioxidant properties, this plant could help fight against the oxidative effects related to cadmium, thereby protecting reproductive function. To our knowledge, no studies have yet been done regarding the use of the ethanolic extract of *Polygonum limbatum meism* (EEPLM) against cadmium-related toxicity in male guinea pigs. However, major investigations are needed for a better understanding of the therapeutic effect of the ethanolic extract of the leaves of *Polygonum limbatum meism* on the harmful effects of cadmium on the reproduction of mammals.

The present study aimed to assess the ability of ethanolic extract of *Polygonum limbatum meism* to fight against cadmium's damages on reproductive characteristics in male guinea pigs.

## Materials and Methods

### Animal Material and Lodging

Thirty-six (36) male adult guinea pigs aged 3–4 months, with a mean body weight of 400 ± 39.15 g were produced at the Teaching and Research Farm of the University of Dschang. The animals were housed in identical cages measuring 100 × 80 × 60 cm (length, width, and height) in well-controlled conditions at room temperature (25.00 ± 2.00°C) and a 12 h light/dark cycle. Experimental protocols used in this study were approved by the Ethical Committee of the Department of Animal Science of the University of Dschang and was in conformity with the internationally accepted standard ethical guidelines for laboratory animal use and care as described in the European Community guidelines, EEC Directive 86/609/EEC, of November 24, 1986.

### Feeding and Chemicals

Animals were fed a basal ration of elephant grass and a supplement of provender. Cadmium chloride was obtained from GEOCHIM SARL Cameroon. The doses of cadmium chloride were chosen from earlier works (dose having the most adverse effects in all studied parameters from our previous study) (14) and were readjusted weekly according to the weight of the guinea pigs. Vitamin C was also obtained from commercial sources (Shalina, Nariman point, Mumbai, India. A/Em/At: Plot No. E-2, M.I.D.C. Jejuri; Tal: Purandar. Dist Pune, Maharashtra, India), and the dose of vitamin C chosen was a clinical dose usually prescribed by veterinarians in the case of disease in animals.

### Plant Extract

Leaves of *Polygonum limbatum meism* (Polygonaceae) were collected from Santchou village near the city of Dschang in February 2020. The plant was identified by the National Herbarium of Cameroon in Yaoundé, where a voucher specimen was deposited under the reference number 38852/HNC. The leaves were dried and crushed to a powder. The dried powdered leaves of *Polygonum limbatum meism* (1,000 × g) were extracted in ethanol (5 L) and allowed to stand at room temperature for 72 h. The percolate was collected and dried in a rotary evaporator at 60°C to obtain the ethanolic extract of *Polygonum limbatum meism* (EEPLM).

The doses of ethanolic extract were chosen from others works that used ethanolic extract (15, 16) and were readjusted weekly according to the weight of guinea pigs. Table 1 shows the bioactive compounds detected using phytochemical tests according to Harborne (17).

**Table 1:** Phytochemical screening of EEPLM leaves.

**Compounds**	**Ethanolic extract**
Phenolic compounds	++
Saponins	+
Steroids	+
Triterpens	+
Flavonoids	++

*+, Present; ++, Highly present*.

### Experimental Design

Animals were divided into six groups of six animals each as follows:

**Group 1:** neutral control: distilled water (1 ml/kg bw).
**Group 2:** negative control: 26.25 mg/kg bw of cadmium chloride.
**Group 3:** positive control: 26.25 mg of cadmium chloride/kg bw and 100 mg of vitamin C/kg bw.
**Group 4:** 26.25 mg of cadmium chloride/kg bw and 50 mg of EEPLM/kg bw.
**Group 5:** 26.25 mg of cadmium chloride/kg bw and 100 mg of EEPLM/kg bw.
**Group 6:** 26.25 mg of cadmium chloride/kg bw and 200 mg of EEPLM/kg bw.

All the animals were treated once a day and every day for 90 days by oral administration because the duration of spermatogenesis in guinea pigs is about 72 days, so by doing 90 days of treatment we were sure that the product could reach all stages of spermatogenesis and the seminal epithelial cycle. Their body weight was recorded weekly and the doses of administered solutions adjusted accordingly. At the end of the trial, animals were sacrificed under ether vapor anesthesia.

### Studied Parameters and Data Collection

#### Reaction Time

A week prior to sacrifice, each animal was housed with an adult female and a chronometer was activated. The chronometer was turned off as soon as reactions including pursuit of the female, descriptive curves around the female, smelling of the ano-genital tract, and attempts to mount were observed from the male, and the time was noted. The maximum observation time for any possible reaction of the male in the presence of a female was 5 min.

#### Serum Testosterone Concentration and Some Biochemical Parameters of Nephrotoxicity and Hepatotoxicity

Blood was collected by cardiac puncture and stored at room temperature. Serum was collected 12 h later (time necessary for the good formation of serum) for the estimation of testosterone concentration using an Omega Diagnostics kit (Scotland, United Kingdom) and alanine and aspartate amino transferases, total cholesterol, total protein, albumin, creatinine, and urea levels, using Chronolab kits (Barcelona, Spain).

#### Oxidative Stress Markers

One of the testes was ground in a 0.9% NaCl solution to obtain 15% homogenates. This was then centrifuged at 3,000 rpm for 30 min, the supernatant was removed and stored at −20°C, and later superoxide dismutase (SOD), total peroxidases (POX), catalase (CAT) activities, and malondialdehyde (MDA) concentration were measured using a spectrophotometer (GENESYS 20.0) and according to the methods described by Misra and Fridovich (18), Habbu et al. (19), Sinha (20), and Nilsson et al. (21), respectively. The testicular total proteins were dosed using a Chronolab kit (Barcelona, Spain).

#### Sexual Organ Weights and Volume of Testes

Ninety days after the treatment, animals were sacrificed and organs including testes, epididymides, vas deferens, and accessory sex glands were removed and weighed. The testicular volumes were determined by immersing these organs in a liquid contained in a graduated burette and by calculating the difference in level (22).

#### Sperm Characteristics

Immediately after dissection, the cauda epididymis was minced using surgical scissors in 5 ml of 0.9% NaCl solution (at 37°C) for sperm concentration, motility, cell membrane integrity, and morphology evaluation. For sperm motility, a drop of the homogenous suspension was placed on a pre-warmed slide, covered with a coverslip, and evaluated under a light microscope at 400× magnification. The motility score was attributed according to Baril et al. (23), using a scale from 0 to 5. The sperm count was evaluated using a Thoma hemocytometer (Thermo Fisher Scientific, Waltham, MA, USA). The count was made under a microscope, at 400× magnification. The spermatozoa were counted in four large squares of the Thoma cell chamber. The operation was performed twice and the average found determined the number of sperm in the four large squares. The percentages of sperm morphological abnormalities (small and big heads, tails winding) and spermatozoa plasma membrane integrity were evaluated using a eosin–nigrosin solution and the hypo-osmotic test, respectively, in a total number of 200 spermatozoa.

#### Histological Sections of Testes

The left testes were fixed in 10.00% formalin for 1 week, then washed, dehydrated with ascending grades of alcohol bath, clarified in xylene immersion, embedded in paraffin, cut at 5.00-μm thickness, and stained with hematoxylin and eosin. The sections were observed under a light microscope (400× magnification).

### Statistical Analysis

Data were analyzed using SPSS IBM statistics software 20.0. Differences among groups were assessed using one-way analysis of variance (ANOVA), followed by Duncan's test at 5% significance. Results were expressed as mean ± standard deviation.

## Results

### Relative Weight and Volume of Reproductive Organs

Vas deferens and the volume of testes (Table 2) decreased significantly (*p* < 0.05) in animals given cadmium chloride only with reference to distilled water-treated animals. Cadmium chloride caused a significant increase (*p* < 0.05) in the weight of the bulbo-urethral gland in guinea pigs exposed only to cadmium chloride compared to that of neutral control animals. However, EEPLM and vitamin C caused a significant decrease (*p* < 0.05) in the weight of the bulbo-urethral gland in animals exposed to cadmium chloride compared to that of animals exposed exclusively to cadmium chloride.

**Table 2:** Effects of EEPLM on reproductive characteristics in male guinea pigs exposed to cadmium chloride.

**Characteristics of reproduction**	**Controls**			**Doses of EEPLM (mg/kg bw)**			***p***
	**DW (*n* = 6)**	**Cd (*n* = 6)**	**VitC (*n* = 6)**	**50 (*n* = 6)**	**100 (*n* = 6)**	**200 (*n* = 6)**	
**Organ weights (g/100 g bw)**							
Testes	0.57 ± 0.04	0.49 ± 0.04	0.53 ± 0.06	0.55 ± 0.04	0.56 ± 0.06	0.57 ± 0.06	0.215
Epididymides	0.12 ± 0.00	0.10 ± 0.00	0.10 ± 0.01	0.11 ± 0.02	0.10 ± 0.01	0.11 ± 0.02	0.201
Seminal vesicle	0.33 ± 0.04^a^	0.26 ± 0.06	0.22 ± 0.07	0.26 ± 0.06	0.25 ± 0.02	0.21 ± 0.06	0.254
Prostate	0.16 ± 0.02	0.12 ± 0.02	0.15 ± 0.03	0.16 ± 0.03	0.16 ± 0.03	0.15 ± 0.03	0.374
Bulbo-urethral Gland	0.029 ± 0.004^b^	0.035 ± 0.002^a^	0.030 ± 0.003^b^	0.029 ± 0.003^bc^	0.028 ± 0.004^bc^	0.024 ± 0.002^c^	0.002
Vas deferens	0.054 ± 0.008^a^	0.039 ± 0.009^b^	0.038 ± 0.010^b^	0.043 ± 0.006^b^	0.036 ± 0.003^b^	0.040 ± 0.001^b^	0.009
Volume of testes (ml)	3.37 ± 0.30^a^	2.85 ± 0.24^b^	2.80 ± 0.14^b^	2.95 ± 0.18^b^	2.90 ± 0.10^b^	3.00 ± 0.18^b^	0.004

*n, number of animals, each value represents mean ± standard error mean. Mean values for each parameter in the same row, with different superscripts (a, b, c) differ significantly (p < 0.05). Cd, 26.25 mg cadmium chloride/kg bw; VitC, 100 mg of vitamin C; DW, distilled water; EEPLM, ethanolic extract of Polygonum limbatum meism. p = probability value*.

### Characteristics of Caudal Epididymal Sperm

The sperm motility, count per cauda epididymis, and plasma membrane integrity (Table 3) declined significantly (*p* < 0.05) in cadmium-treated guinea pigs, compared to the control receiving distilled water. The treatment of animals exposed to cadmium with EEPLM showed an increase in values of those sperm characteristics, but the difference was significant (*p* < 0.05) just for sperm motility and plasma membrane integrity at the dose of 50 mg of EEPLM as compared to animals submitted to cadmium only. In contrast, the percentages of abnormal spermatozoa (micro and macrocephalies and coiled tails) (Table 3) increased significantly (*p* < 0.05) in cadmium-treated males than the control receiving distilled water. The administration of plant extract whatever the dose led to a significant (*p* < 0.05) decrease in animals exposed to cadmium compared to animals exposed to cadmium chloride only (Table 2).

**Table 3:** Effects of EEPLM on epididymal spermatozoa characteristics in guinea pigs exposed to cadmium chloride.

**Epididymal spermatozoa characteristics**	**Controls**			**Doses of EEPLM (mg/kg bw)**			***p***
	**DW (*n* = 6)**	**Cd (*n* = 6)**	**VitC (*n* = 6)**	**50 (*n* = 6)**	**100 (*n* = 6)**	**200 (*n* = 6)**	
Motility (%)	76.67 ± 8.17^ab^	62.50 ± 5.00^c^	67.50 ± 9.6^bc^	78.00 ± 8.36^ab^	80.00 ± 10.00^ab^	82.00 ± 10.95^a^	0.028
Count per tail (×10^7^)	8.94 ± 1.32^a^	6.13 ± 0.27^b^	6.58 ± 1.74^b^	7.15 ± 0.78^ab^	6.75 ± 0.88^b^	7.19 ± 1.90^ab^	0.040
Count per g of tail (×10^7^)	31.87 ± 7.50	23.65 ± 3.74	24.82 ± 3.62	26.50 ± 6.49	29.45 ± 6.86	25.07 ± 7.05	0.324
Spermatozoa with EPM (%)	71.87 ± 3.08^a^	59.50 ± 4.25^c^	69.78 ± 7.23^ab^	68.90 ± 5.27^ab^	67.42 ± 8.61^abc^	61.61 ± 5.83^bc^	0.016
Coiled tails (%)	0.20 ± 0.11^cd^	0.71 ± 0.19^a^	0.66 ± 0.21^a^	0.34 ± 0.20^cd^	0.00 ± 0.00^d^	0.48 ± 0.46^ab^	0.001
Micro and macro cephalies (%)	0.51 ± 0.15^b^	1.18 ± 0.45^a^	0.34 ± 0.12^b^	0.64 ± 0.29^b^	0.55 ± 0.19^b^	0.50 ± 0.19^b^	0.002

*N, number of animals, each value represents mean ± standard error mean. Mean values for each parameter in the same row, with different superscripts (a, b, c) differ significantly (p < 0.05). Cd, 26.25 mg cadmium chloride/kg bw; VitC, 100 mg of vitamin C; DW, distilled water. EEPLM, ethanolic extract of Polygonum limbatum meism. p = probability value*.

### Biochemical Parameters

Serum levels of ALT, AST, creatinine, and urea (Table 4) were significantly (*p* < 0.05) increased in animals poisoned with cadmium chloride compared to those in animals force-fed with distilled water. Ethanolic extracts as well as vitamin C significantly reduced (*p* < 0.05) the serum levels of ALT, AST, creatinine, and urea in guinea pigs exposed to the heavy metal compared to animals treated exclusively with cadmium chloride.

**Table 4:** Effects of EEPLM on toxicity biomarkers in male guinea pigs exposed to cadmium chloride.

**Toxicity biomarkers**	**Controls**			**Doses of EEPLM (mg/kg bw)**			***p***
	**DW (*n* = 6)**	**Cd (*n* = 6)**	**VitC (*n* = 6)**	**50 (*n* = 6**	**100 (*n* = 6)**	**200 (*n* = 6)**	
ALT (UI)	56.29 ± 9.64^d^	143.06 ± 20.19^a^	94.68 ± 14.92^c^	107.45 ± 11.69^bc^	125.78 ± 15.42^b^	101.85 ± 13.65^c^	0.000
AST (UI)	91.35 ± 13.19^b^	133.88 ± 19.85^a^	109.90 ± 19.93^b^	108.85 ± 9.63^b^	113.53 ± 14.41^b^	92.05 ± 14.04^b^	0.003
Serum total protein (g/dl)	5.19 ± 0.78	4.68 ± 0.45	5.09 ± 0.57	4.98 ± 0.73	5.22 ± 0.60	5.10 ± 0.52	0.645
Albumin (g/dl)	2.93 ± 0.40	2.80 ± 0.18	2.97 ± 0.25	2.66 ± 0.40	2.67 ± 0.50	2.74 ± 0.46	0.501
Globulin (g/dl)	2.26 ± 0.46	1,89 ± 0,33	2.12 ± 0.67	2.32 ± 0.71	2.55 ± 0.37	2.36 ± 0.64	0.410
Serum total cholesterol (mg/dl) (mg/dl)	41.36 ± 4.74	36.81 ± 3.88	42.34 ± 5.52	39.54 ± 6.67	40.23 ± 4.22	37.97 ± 2.81	0.395
Creatinine (mg/dl)	0.80 ± 0.30^b^	1.16 ± 0.31^a^	0.70 ± 0.21^b^	0.96 ± 0.37^ab^	0.80 ± 0.19^b^	0.79 ± 0.17^b^	0.151
Serum urea (mg/dl)	49.03 ± 7.58^c^	82.27 ± 18.99^a^	68.94 ± 10.12^ab^	62.50 ± 9.81^bc^	49.72 ± 9.48^c^	67.42 ± 10.36^ab^	0.000

*n, number of animals, each value represents mean ± standard error mean. Mean values for each parameter in the same row, with different superscripts (a, b, c) differ significantly (p < 0.05). Cd, 26.25 mg cadmium chloride/kg bw; VitC, 100 mg of vitamin C; DW, distilled water; EEPLM, ethanolic extract of Polygonum limbatum meism; ALT, alanine aminotransferase; AST, aspartate aminotransferase; p = probability value*.

Testicular activity of SOD, POX, and MDA level were significantly (*p* < 0.05) elevated in animals exposed to cadmium chloride compared to guinea pigs given distilled water.

### Testosterone Concentration and Reaction Time (Libido)

**Figure 1:**
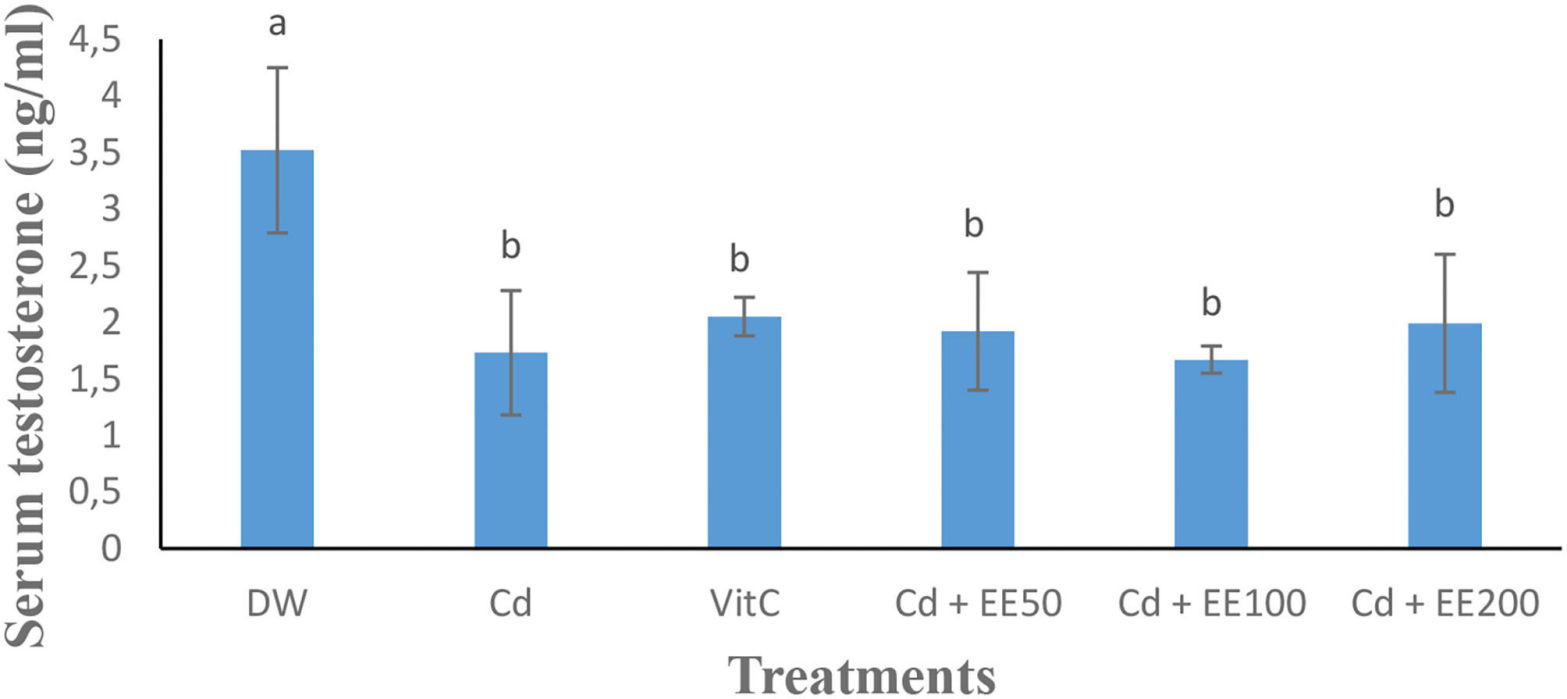
Effects of different concentrations of ethanolic extract of *Polygonum limbatum meism* (EEPLM) on serum testosterone level in male guinea pigs exposed to cadmium chloride. a, b: histogram values with the same letters are not significantly (*p* > 0.05) different.

**Figure 2:**
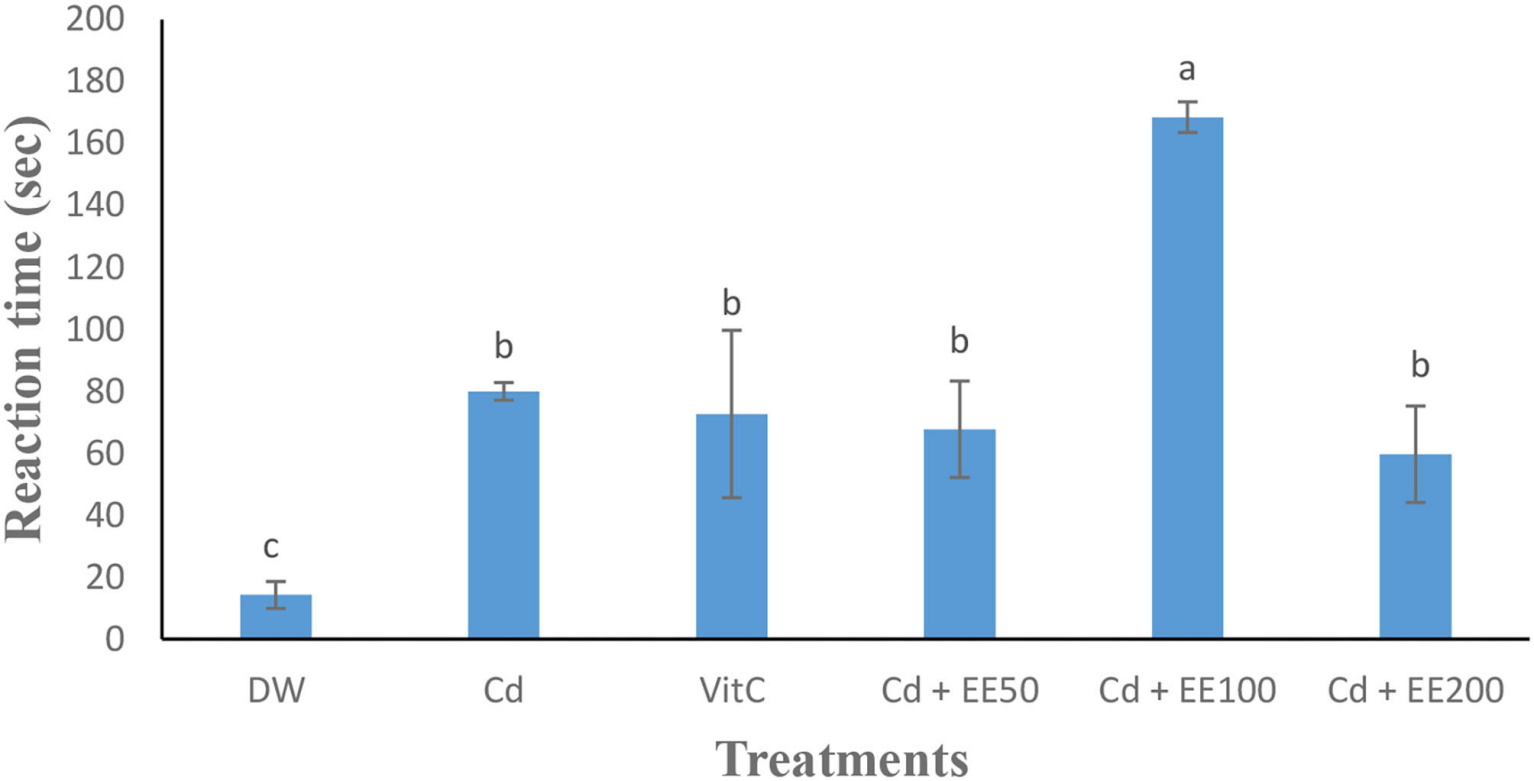
Effects of different concentrations of ethanolic extract of *Polygonum limbatum meism* (EEPLM) on reaction time in male guinea pigs exposed to cadmium chloride. a, b, c: histogram values with the same letters are not significantly (*p* > 0.05) different.

The testosterone level (Figure 1) was significantly (*p* < 0.05) low in the animals exposed to cadmium chloride compared to the guinea pigs receiving distilled water. Cadmium chloride caused a significant (*p* < 0.05) increase in reaction time in guinea pigs compared to neutral control animals. The reaction time was significantly (*p* < 0.05) longer in the group treated at the 100 mg/dose kg bw of ethanolic extract compared to animals receiving distilled water (Figure 2).

### Histological Structure of Testes

**Figure 3:**
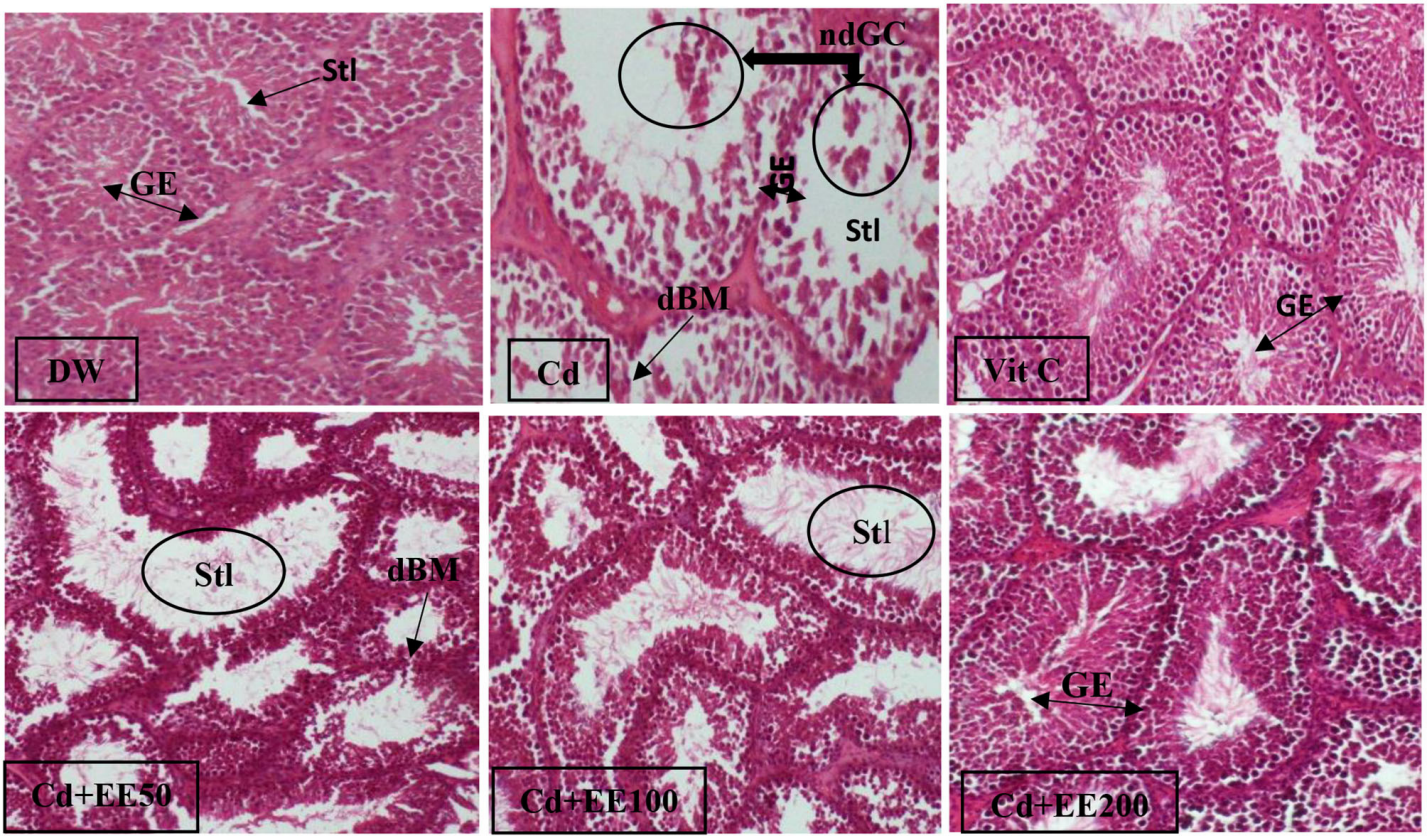
Histological sections of testes in guinea pigs exposed to cadmium chloride and treated with EEPLM leaves (HE × 400). dBM, degraded basal membrane; ndGC, non differentiated germinal cells; Stl, seminiferous tubule lumen; GE, germinal epithelium. DW, neutral control (distilled water); Cd, negative control (26.25 mg of cadmium chloride/kg bw); VitC, positive control (26.25 mg of cadmium chloride/kg bw and 100 mg/kg bw of vitamin C); Cd + EE50, Cd + EE100, and Cd + EE200: (50, 100, 200 mg/kg bw of EEPLM leaves and 26.25 mg of cadmium chloride/kg bw).

Figure 3 illustrates the effect of ethanolic extracts from the leaves of *Polygomome lymbatum meism* on the histological structures of the testis in male guinea pigs exposed to cadmium chloride. In animals given distilled water, the arrangement of basement membrane cells in the lumen of the seminiferous tubules was normal with the presence of mature sperm in the lumen. This arrangement was disrupted in animals treated with cadmium chloride, whose histological sections revealed immature germ cells in the lumen of the seminiferous tubule and destruction of the basement membrane. However, in guinea pigs co-treated with cadmium chloride and ethanolic extracts of leaves of *Polygonum limbatum meism*, almost all disturbances were restored, including the absence of immature germ cells in the lumen of the seminiferous tubules. The same observation was made in animals co-treated with cadmium chloride and vitamin C.

## Discussion

Reproductive toxicity of cadmium chloride in male guinea pigs was characterized by reduced weights of vas deferens and volume of testes in this study. Ren et al. (24) and Olaniyi et al. (25) also reported decreased reproductive organ weight in rats exposed to CdCl_2_ (5 mg/kg; 30 days) and in mice exposed to CdCl_2_ (8 mg/kg; 8 days), respectively. This could also be justified by the fact that a dysfunction of the kidneys or the liver as observed in this study through the abnormal levels of their markers could have an influence on the hypothalamo-pituitary complex and lead to a disruption of the gonadal hormones, in particular the decrease in blood testosterone levels in a renal failure case (26) and a decrease in the production of cholesterol which is a major precursor of testosterone in a hepatic dysfunction case. As testosterone is responsible for the development of the reproductive organs, its low level would consequently lead to a decrease in the weight and volume of the reproductive organs observed in this study. Treatment of male guinea pigs with a combination of EEPLM and cadmium chloride attenuated the cadmium-induced impairment of reproductive parameters. These results indicate that EEPLM might have a beneficial effect in reducing cadmium toxicity. This observation might be explained by the action of antioxidant compounds such as phenols, especially phycocianin, flavonoids, xanthons, terpenoids, and anthaquinons (27, 28) present in *Polygonum limbatum* extract. In this study, cadmium chloride caused a significant increase (*p* < 0.05) in the weight of the bulbo-urethral gland in guinea pigs exposed to cadmium chloride. This increase in the weight of the bulbo-urethral gland could be due to the hyperactivity of this gland in order to eliminate the harmful effects due to cadmium, hence the increase in its weight because the more a cell is in activity, the higher its volume, and that weight could increase. However, EEPLM and vitamin C caused a significant decrease (*p* < 0.05) in the weight of the bulbo-urethral gland in animals exposed to cadmium chloride compared to that of animals exposed exclusively to cadmium chloride. This result could be justified by the presence in EEPLM of antioxidant elements such as phenols and flavonoids capable of neutralizing the free radicals induced by cadmium chloride.

In this study, the damage observed on characteristics of sperm in cadmium chloride-treated animals could be explained by the changes in the properties of the plasma membrane of sperm as a result of the overproduction of reactive oxygen species (29) induced by the cadmium. In fact, spermatozoa plasma membranes are made up of phospholipid bilayers, very rich in unsaturated fatty acids. Therefore, they are susceptible to attack by free radicals, leading to their damage. In addition, this could be due to an impaired follicle-stimulating hormone (FSH) metabolism at the testicular level (30), since spermatogenesis is under the control of this hormone. However, the co-treatment of cadmium and EEPLM improved the sperm motility, count, spermatozoa with entire plasma membrane, and percentage of abnormal spermatozoa. These results are similar to those of Akunna et al. (31) in which the co-administration of chromium (150 mg/kg bw) with *Moringa olifera* (60 mg/kg bw) for 100 days led to the improvement of spermatozoa characteristics. The increase in sperm count, motility, and plasma membrane integrity and decrease of the percentage of abnormal spermatozoa in this study could be due to the antioxidant elements present in the ethanol extract of *Polygonum limbatum* leaves. This result could be justified also by the presence in this plant of antioxidant elements such as phenols and flavonoids capable of neutralizing the free radicals induced by cadmium, thereby attenuating the harmful effects due to this metal.

In this study, administration of cadmium chloride resulted in a decrease in serum testosterone concentration. These results are in agreement with those of Çilenk et al. (32) who reported that exposure of male rats to cadmium chloride (1 mg/kg/day) results in a decrease in serum testosterone levels. The decrease in the production of this hormone could be due to an inhibition of testicular enzymes involved in the biosynthesis of testosterone (33) and could be due to the drop in cholesterol observed in this study. In fact, the cholesterol produced mainly by the liver is one of the major precursors of testosterone. Ethanolic extract of *Polygonum limbatum meism* allowed for an increasing testosterone level in heavy metal-treated guinea pigs compared to control animals force-fed with distilled water. These results are similar to those of Çilenk et al. (32) who reported that exposure of male rats to cadmium chloride (1 mg/kg/day) and ethanolic propolis extract (50 mg/kg/day) orally for 17 days resulted in an increase in serum testosterone levels compared to controls receiving only the heavy metal. This could be due to the presence of antioxidant elements in the leaves of *Polygonum limbatum meism* which will help neutralize the overproduction of free radicals responsible for the destruction of cells responsible for the secretion of testosterone and thus promote their production in guinea pigs exposed to cadmium chloride.

The reaction time was short in animals co-administered with cadmium chloride and ethanolic extract of *Polygonum lymbatum meism* compared to guinea pigs exposed only to cadmium chloride. These results are similar to those of Guiekep et al. (16) who showed that a co-administration of acetamiprid at doses of 80 mg/kg and ethanolic extract of leaves of *Mangifera indica* at doses of 50, 100, and 200 mg/kg for 90 days made it possible to reduce the reaction time of male guinea pigs in the presence of a female compared to animals that received only acetamiprid. This could be due to the increase in testosterone observed in these animals co-treated with cadmium chloride and ethanolic extract of *Polygonum limbatum* and also by the presence of androgenic molecules in the leaves of *Polygonum limbatum meism* thus playing the same role as testosterone and consequently helping to reduce the reaction time in these metal-poisoned animals.

In the present study, the arrangement of basement membrane cells in the lumen of the seminiferous tubules was disrupted in animals treated with cadmium chloride only, whose histological sections revealed immature germ cells in the lumen of the seminiferous tubule and destruction of the basement membrane. These results are in accordance with those of De Souza Predes et al. (34) observed in rats injected intraperitoneally with cadmium chloride (1.2 mg/kg bw). This damage could be justified by the fact that the defenses of the testes against cadmium contamination are possibly a function of the level of metallothionein (MT) present in the testes. It is widely believed that cadmium bound to metalothionein is non-toxic. Thus, metallothioneins protect tissues against the toxicity of cadmium. In this way, the balance between Cd-MT and free Cd in tissues has been shown to be of crucial importance for toxicity (35). In addition, Xu et al. (35) and Siu et al. (36) claim that Cd treatment induces metallothionein production, but this appears to be limited to a certain level in the testes. Toxic effects probably result from the fact that the amount of MT becomes insufficient to bind to the amount of Cd present in the testicular cells leading to oxidative stress and consequently structural alteration (35). However, in guinea pigs co-treated with cadmium chloride and ethanolic extracts of the leaves of *Polygonum limbatum meism*, almost all disturbances were restored, including the absence of immature germ cells in the lumen of the seminiferous tubules. This result could be justified by the presence in this plant of antioxidant elements such as phenols and flavonoids capable of neutralizing the free radicals induced by cadmium, thereby attenuating the harmful effects due to this metal on the testicular structures.

Hepatocellular enzymes (ALT, AST) and the levels of total proteins, cholesterol, creatinine, and urea are used to evaluate the function of the liver and kidney. The decrease in their activities in the liver and the kidney or their increased level in blood could be associated with the pathology involving necrosis of these organs. In this study, the low level of cholesterol could be attributed to the damaging effects of cadmium chloride on liver and kidney cells as confirmed by the increase in the activities of serum ALT, AST, creatinine, and urea. However, administration of EEPLM allowed these parameters to restore toward normal, which may be due to its anti-oxidative and hepato-protective activities that scavenged the reactive oxygen species due its content. *Polygonum limbatum* is an important herbal medicine that has antioxidant properties and scavengers of free radicals (28).

In this study, the level of MDA, SOD, catalase, and total peroxidase activities (Table 5) decreased significantly and became normal in animals co-administered with cadmium chloride and EEPLM compared to the negative control receiving cadmium only. These results are similar to those of Deutcheu et al. (15) who reported the same results in lead acetate-treated guinea pigs for the first 30 days at the dose 12 mg/kg bw and then administered 100 mg/kg bw of hydroethanolic extract of *Spirulina platensis* from the 31st day to the 90th day. Nevertheless, restoration of those stress parameters in cadmium chloride and EEPLM-treated animals suggested that *Polygonum limbatum meism* could have protective effects against oxidative stress induced by cadmium chloride (37). However, in this study, contrary to the results observed in Deutcheu et al. (15), the activity of antioxidant enzymes rather increased in animals exposed to metal only. These results could be justified by the fact that when the stress is moderate, the organism will try to react by over-expressing the level of antioxidant enzymes to try to fight this stress (38, 39).

**Table 5:** Effects of EEPLM on oxidative stress biomarkers in male guinea pigs exposed to cadmium chloride.

**Characteristics of reproduction**	**Controls**			**Doses of EEPLM (mg/kg.bw)**			***p***
	**DW (*n* = 6)**	**Cd (*n* = 6)**	**VitC (*n* = 6)**	**50 (*n* = 6**	**100 (*n* = 6)**	**200 (*n* = 6)**	
SOD (μM/min/g)	0.19 ± 0.03^bc^	0.35 ± 0.07^a^	0.16 ± 0.04^c^	0.25 ± 0.09^b^	0.19 ± 0.09^bc^	0.23 ± 0.09^bc^	0.001
CAT (μM/min/g)	1.09 ± 0.2	1.29 ± 0.2	1.08 ± 0.32	1.04 ± 0.21	1.18 ± 0.37	1.16 ± 0.11	0.437
MDA (μM)	1.48 ± 0.18^b^	2.14 ± 0.28^a^	1.54 ± 0.26^b^	1.67 ± 0.12^b^	1.50 ± 0.36^b^	1.55 ± 0.42^b^	0.001
POX (μM/min/g)	24.3 ± 5.55^b^	35.73 ± 9.32^a^	27.7 ± 9.32^b^	28.05 ± 4.47^b^	31.1 ± 3.63^ab^	28.20 ± 6.24^b^	0.040
TTproteins (g/dl)	2.13 ± 0.09^ab^	2.02 ± 0.09^b^	2.20 ± 0.09^a^	2.15 ± 0.12^ab^	2.01 ± 0.09^b^	2.11 ± 0.2^b^	0.020

*n, number of animals, each value represents mean ± standard error mean. Mean values for each parameter in the same row, with different superscripts (a, b, c) differ significantly (p < 0.05). Cd, 26.25 mg cadmium chloride/kg bw; VitC, 100 mg of vitamin C; DW, distilled water; EEPLM, ethanolic extract of Polygonum limbatum meism; SOD, superoxide dismutase; CAT, catalase; POX, peroxidase glutathione; MDA, malondialdehyde; TTproteins, testicular total proteins. p = probability value*.

## Conclusion

It can be concluded that cadmium chloride induced oxidative stress, which negatively affected the reproductive function in male guinea pigs. However, treatment with *Polygonum limbatum meism* extract fights against the action of free radicals and ameliorates reproductive characteristics. Thus, the ethanolic extract of *Polygonum limbatum meism* as well as vitamin C could be used as a curative alternative to alleviate the effects of reproductive stress induced by cadmium chloride on the male reproductive system. In the case of its utilization, the dose 200 mg/kg bw is recommended.

## Data Availability Statement

The raw data supporting the conclusions of this article will be made available by the authors, without undue reservation.

## Ethics Statement

Experimental protocols used in this study were approved by the Ethical Committee of the Department of Animal Science of the University of Dschang and was in conformity with the internationally accepted standard ethical guidelines for laboratory animal use and care as described in the European Community guidelines; EEC Directive 86/609/EEC, of November 24, 1986.

## Author Contributions

AK: supervised and designed the project, cross checked the draft of the manuscript, and finally approved for submission. BM, BV, and AK: designed the project, conducted the experiment, collected, analyzed data, and wrote the first draft of the manuscript. MT, ND, TF, JB, and MC: assisted in the conduction of the experiment and collected data. BV and ND: conducted laboratory analysis of experiment. BV and MC: rechecked the draft of the manuscript. All authors contributed to the article and approved the submitted version.

## Conflict of Interest

The authors declare that the research was conducted in the absence of any commercial or financial relationships that could be construed as a potential conflict of interest.

## Publisher's Note

All claims expressed in this article are solely those of the authors and do not necessarily represent those of their affiliated organizations, or those of the publisher, the editors and the reviewers. Any product that may be evaluated in this article, or claim that may be made by its manufacturer, is not guaranteed or endorsed by the publisher.
